# Biological Properties of Iron Oxide Nanoparticles for Cellular and Molecular Magnetic Resonance Imaging

**DOI:** 10.3390/ijms12010012

**Published:** 2010-12-23

**Authors:** Thomas Schlorf, Manuela Meincke, Elke Kossel, Claus-Christian Glüer, Olav Jansen, Rolf Mentlein

**Affiliations:** 1 Department of Anatomy, Christian-Albrechts-University Kiel, Otto-Hahn-Platz 8, 24118 Kiel, Germany; E-Mails: thomas.schlorf@gmx.de (T.S.); m.meincke@anat.uni-kiel.de (M.M.); 2 Department of Radiology, Division of Medical Physics, University Clinic Schleswig-Holstein, Campus Kiel, Arnold Heller Straße 9, 24105 Kiel, Germany; E-Mails: ekossel@ifm-geomar.de (E.K.); ccglueer@web.de (C.-C.G.); 3 Department of Neuroradiology, University Clinic Schleswig-Holstein, Campus Kiel, Schittenhelmstraße 10, 24105 Kiel, Germany; E-Mail: o.jansen@neurorad.uni-kiel.de

**Keywords:** iron oxide nanoparticles, magnetic resonance imaging (MRI), toxicity, uptake kinetics, cellular localization, iron staining, electron microscopy

## Abstract

Superparamagnetic iron-oxide particles (SPIO) are used in different ways as contrast agents for magnetic resonance imaging (MRI): Particles with high nonspecific uptake are required for unspecific labeling of phagocytic cells whereas those that target specific molecules need to have very low unspecific cellular uptake. We compared iron-oxide particles with different core materials (magnetite, maghemite), different coatings (none, dextran, carboxydextran, polystyrene) and different hydrodynamic diameters (20–850 nm) for internalization kinetics, release of internalized particles, toxicity, localization of particles and ability to generate contrast in MRI. Particle uptake was investigated with U118 glioma cells und human umbilical vein endothelial cells (HUVEC), which exhibit different phagocytic properties. In both cell types, the contrast agents Resovist, B102, non-coated Fe_3_O_4_ particles and microspheres were better internalized than dextran-coated Nanomag particles. SPIO uptake into the cells increased with particle/iron concentrations. Maximum intracellular accumulation of iron particles was observed between 24 h to 36 h of exposure. Most particles were retained in the cells for at least two weeks, were deeply internalized, and only few remained adsorbed at the cell surface. Internalized particles clustered in the cytosol of the cells. Furthermore, all particles showed a low toxicity. By MRI, monolayers consisting of 5000 Resovist-labeled cells could easily be visualized. Thus, for unspecific cell labeling, Resovist and microspheres show the highest potential, whereas Nanomag particles are promising contrast agents for target-specific labeling.

## 1. Introduction

Small supraparamagnetic iron oxide particles are increasingly used in magnetic resonance imaging (MRI) either as direct contrast agents *in vivo*, as conjugates for monitoring specific molecules *in vitro* and *in vivo*, or for *ex vivo*-labeling of cells and tracking their fate *in vivo* [1,2]. As compared with (non-polymeric) gadolinium complexes, magnetic iron oxide particles appear to yield higher detection sensitivities [3,4], but have the disadvantage for molecular imaging (or the advantage to monitor phagocytic cells) to be rapidly internalized by phagocytosis. To stabilize iron oxide particles in solution and to reduce their non-specific uptake, they are commonly coated by dextran derivatives. However, little is known about how these modifications influence toxicity, phagocytosis and cellular retention of these particles.

Therefore, we compared these properties for several supraparamagnetic particles with magnetite and maghemite cores and different coatings. For these studies, we used different human cell types, namely glioma cells that are characterized (as many other tumor cells) by a high phagocytic potential [5], as well as endothelial cells from human umbilical vein endothelial cells (HUVEC) with a supposed lower phagocytosis [6]. We analyzed the internalization kinetics of the particles, their cellular localization after uptake, their *in vitro* toxicity, and finally their contrast properties *in vitro* under the aspects of suitability for *ex vivo* cellular labeling and/or for selective molecular imaging for *in vivo* MRI visualization.

## 2. Results and Discussion

### 2.1. Comparison of Particle Internalization and Internalization Kinetics *in Vitro*

Iron-oxide particles with different core materials (magnetite, maghemite), different coatings (none, dextran, carboxydextran, polystyrene) and different hydrodynamic diameters (20–850 nm) were chosen (Table 1) and their internalization was monitored initially after 12, 24, 36 and 48 h in U118 glioma cells and HUVEC.

**Figure 1:**
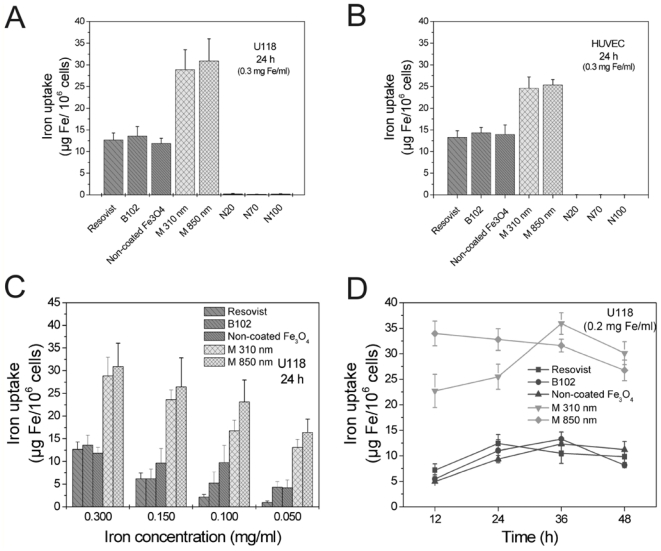
Internalization of different iron oxide nanoparticles (see Table 1) in human U118 glioma cells (**A**, **C**, **D**) and human umbilical vein endothelial cells (HUVEC, B) after 24 h at an iron concentration of 0.3 mg/mL; (**A**, **B**) The microspheres (0.31 μm and 0.85 μm) were absorbed best, Resovist, B102 and non-coated Fe_3_O_4_ showed an equally strong high uptake, whereas NanomagN20, N70 and N100 were not measurably internalized; (**C**) Iron uptake after 24 h as a function of the iron/particle concentrations applied; (**D**) Different uptake kinetics were observed for the different particles at the same iron concentration (0.2 mg Fe/mL); however, the strongest uptake was mostly observed between 24 and 36 h; (**A**–**D**) *n* = 3 ± S.D.

In both cell lines, Resovist, B102, the non-coated Fe_3_O_4_ and the two microspheres were strongly absorbed, whereas Nanomag20, Nanomag70 and Nanomag100 showed a low iron uptake into the cells (Figures 1A and 1B).

After 24 h, uptake of nanoparticles was dependent on the iron concentration in the culture medium (Figure 1C). The uptake kinetics differed somewhat between the nanoparticles, however, maximal uptake was mostly observed between 24 h through 36 h exposure at an iron concentration of 0.2 mg/mL (Figure 1D). Afterwards, iron content within the cells decreased slightly. Therefore, all subsequent cell-labeling experiments were performed with an incubation time of 24 h. To get deeper insight into reasons for iron particle loss after maximum absorption, we investigated the long-term stability of cell-labeling and the cytotoxicity of the particles.

### 2.2. Long-Term Stability of Labeling *in Vitro*

**Figure 2:**
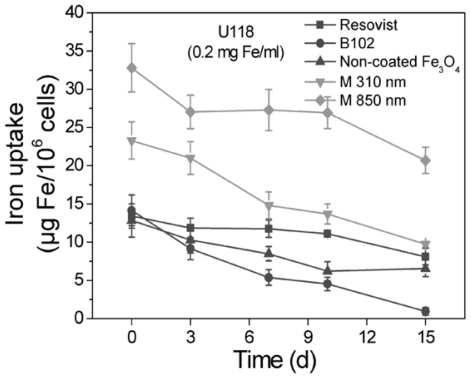
Long term stability of cell-labeling by nanoparticles. U118 glioma cells were incubated with different iron oxide nanoparticles for 24 h at an iron concentration of 0.2 mg/mL, washed and further cultivated. The cellular iron content was measured after different periods of time. Resovist showed the longest resting time, B102 the shortest. Initial iron concentration varied due to different initial labeling efficiencies, see Figure 1. *n* = 3 ± S.D.

The long-term stability of labeling is indispensable for subsequent cell tracking experiments. Therefore, cells were labeled with Resovist, B102, the non-coated Fe_3_O_4_, Microsphere (0.31) and Microsphere (0.85) as in previous experiments (24 h and 0.2 mg Fe/mL). After 24 h, the medium was replaced, the cells were cultivated for a maximum time of two weeks and the iron content in the cells was determined (Figure 2). Of all nanoparticles investigated, Resovist showed the least iron release; despite the fact that the iron concentration inside the cells was higher for microsphere-labeled cells due to their strong iron uptake during the 24 h incubation period. Iron release from Fe_3_O_4_ labeled cells was higher than the release from Resovist labeled cells but still smaller than the release from B102 labeled cells.

### 2.3. Cellular Toxicity and Plasma Binding

The cellular toxicity of iron oxide nanoparticles *in vitro* was determined by measuring the release of the cytosolic enzyme lactate dehydrogenase (LDH) into the cell culture supernatants. Like in previous experiments, the iron concentration of all particles was 0.2 mg Fe/mL and the incubation period was 24 h. As control for complete LDH release, one sample was lysed and its LDH value taken as 100% toxicity.

**Figure 3:**
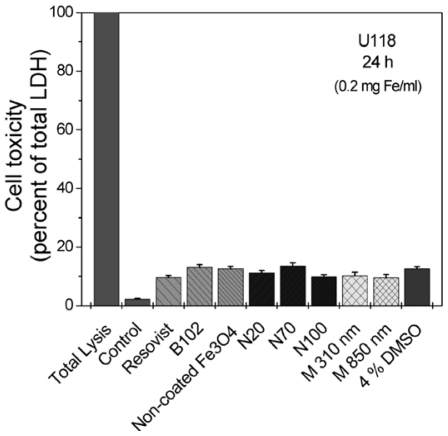
Toxicity of the particles as measured by the release of the cytosolic enzyme lactate dehydrogenase (LDH) *in vitro*. U118 cells were exposed to nanoparticles for 24 h at an iron concentration of 0.2 mg/mL at 1% fetal calf serum (FCS, heat-inactivated), and released LDH was quantified in the culture supernatants; LDH-activity in lysed cells yielded the total activity (100%). All particles produced only a slight LDH-release comparably to the effect of 4% dimethyl sulfoxide (DMSO). *n* = 3 ± S.D.

At 1% fetal calf serum (FCS) in the culture dishes, all iron oxide nanoparticles effected only a slightly release of LDH in the culture medium (Figure 3). The effect was comparable to the exposure to 4% dimethyl sulfoxide (DMSO).

**Figure 4:**
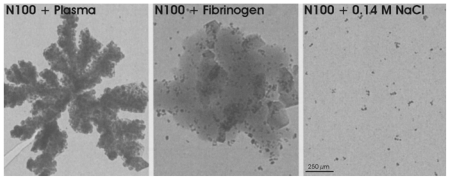
Aggregation of N100-particles after 1 h exposure to human plasma, human fibrinogen (0.21 mg/mL) or phosphate-buffered saline (control), revealed by electron microscopy.

However, exposure of some particles (e.g., N100) to human plasma or to fibrinogen resulted in an aggregation of the particles (Figure 4). This should be considered when they are injected *in vivo.*

### 2.4. Cellular Localization of Absorbed Iron Oxide Nanoparticles

**Figure 5:**
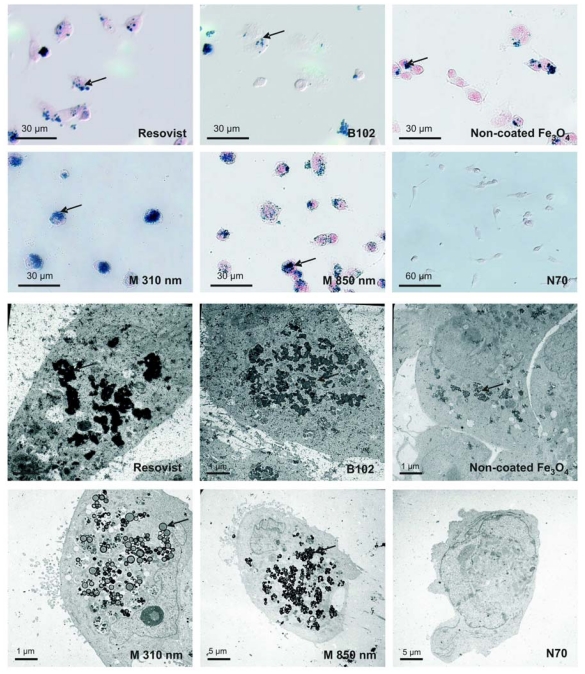
Visualization of the internalized particles by light microscopy after Prussian blue staining (top) and electron microscopy (bottom). U118 glioma cells were exposed to the particles for 24 h at 0.2 mg iron/mL. All particles were internalized (examples marked with arrows) except Nanomag-particles N20, N70 and N100. Resovist, B102 and non-coated Fe_3_O_4_ clustered in the cells but the microspheres (M 850 und M 310) did not. Typical results from three individual incubations are shown for each.

To clarify whether the nanoparticles were adsorbed at the cell surface or internalized, we visualized them by light microscopic Prussian Blue staining and by electron microscopy. The cells were incubated with different iron oxide nanoparticles (0.2 mg Fe/mL) over 24 h. In particular, the electron microscopy images clearly displayed that the nanoparticles were located mostly intracellular and only rarely at the cell surface (Figure 5, bottom). The Prussian Blue Staining pictures sustained this impression (Figure 5, top). In accordance to the iron measurements, highest labeling was achieved with both the microspheres and no labeling was seen with the Nanomag particles. Whereas microspheres did not form clusters inside the cells, B102, Resovist and the non-coated Fe_3_O_4_ yielded large clusters diffusely distributed in the cells. Prussian blue staining or electron microscopy could not reveal a confinement of the clusters by specific cell organelles.

### 2.5. MRI Visualization *in Vitro*

To compare the efficiency of the labeling for MRI visualization, cells were cultivated as monolayers on polyethylene terephthalate (PET) membrane filters [7]. These give only a negligible contrast in MRI and for this reason they are also only slightly visible. Between 1000 and 100,000 cells were seeded on the membranes and incubated with different iron oxide nanoparticles at 0.2 mg iron/mL over 24 h. The internalized iron oxide nanoparticles were imaged with a T2*-weighted pulse sequence on a 3 Tesla MRI system (Philips, Achieva).

**Figure 6:**
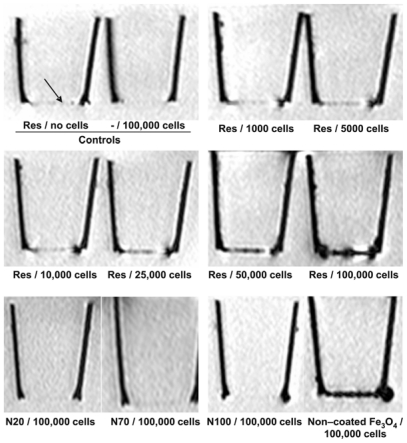
MRI visualization of nanoparticles-labeled U118 glioma cells grown as a monolayer on membrane filters. Cells grown on polyethylene terephthalate (PET) membrane filters were labeled for 24 h with nanoparticles (0.2 mg Fe/mL) and washed. T2*-weighted images were generated with a 3 Tesla MRI at room temperature. Controls with Resovist or 100,000 unlabelled cells are negative (arrow). After labeling with Resovist, already 5000 cells caused a visible signal reduction. Similar results were obtained with microspheres (850 und 310 nm), B102 and non-coated Fe_3_O_4_ (shown only in one example). In contrast, Nanomag particles N20, N70 and N100 yielded no signals, even at high cell numbers. Typical results from three individual incubations are shown for each.

Controls, namely cell free membranes that were incubated with the nanoparticles alone and membranes with cells, but without nanoparticle incubation, yielded no signals (Figure 6). In contrast, cells incubated with the nanoparticles Resovist, microspheres (850 and 310 nm), B102 and non-coated Fe_3_O_4_ showed a clearly visible labeling. Cells incubated with the nanoparticles N20, N70 and N100 were not detectable (Figure 6, bottom). To determine the detection threshold, we applied Resovist to different numbers of cells. We could show that as few as 5000 labeled cells produced a visible difference to both control membranes (Figure 6, top); a higher cell numbers yielded proportionally higher contrasts (Figure 6, center row). Thus, iron oxide nanoparticles that are highly internalized exhibit a high sensitivity for detection of the cells in MRI scanners; furthermore, the MRI signal reduction corresponds to the iron concentrations within the cells.

## 3. Experimental Section

### 3.1. Cell Culture, Contrast Agents and Cell Labeling

The human glioma cell line U118 (obtained from the Deutsche Krebsforschungsinstitut, Heidelberg, Germany) was cultured in Dulbecco’s modified Eagle’s medium (DMEM) supplemented with 10% fetal calf serum (FCS). Human umbilical vein epithelial cells (HUVEC) were cultured in endothelial cell basal medium (PromoCell, Heidelberg, Germany) supplemented with 5% FCS, 0.1 ng/mL human epidermal growth factor (hEGF), 4% heparin and 1 ng/mL human basic fibroblast growth factor (hbFGF) under standard culture conditions (5% CO_2_, 95% air in humidified chamber at 37 °C) and split every 3 days as detailed elsewhere [8,9].

Contrast agents were obtained from the suppliers listed in Table 1. The different iron oxide particles were added to the respective media described before. After incubation, cells were washed twice briefly with phosphate buffered saline (PBS) and the iron uptake was quantified by photometrical determination (see below) Alternatively, internalized iron was visualized by Prussian Blue Staining, electron microscopy or MRI (see below). Unlabeled cells served as controls.

### 3.2. Quantification of Iron

Cells were dissolved in 1 mL 37% hydrochloric acid (HCl) and heated at 70 °C for 10 min. After cooling down to room temperature, 0.1 mL-aliquots were mixed with 4.9 mL of 0.68 M citrate-0.64 M phosphate buffer, pH 3.1. Then, 0.1 mL of these dilutions were incubated in a 96-well plate with 0.1 mL Spectroquant-reagent (Merck, Darmstadt, Germany; code 1.1476, 1.0001 containing thioglycolic acid to reduce Fe^3+^ to Fe^2+^ and the chromogenic reagent Na_2_[3-(2-pyridyl)-5,6-bis(4- phenylsulfonic acid)-1,2,4-triazine]) diluted 1:50 in the above buffer. After 30 min, the bathochromic shift was measured by an Elisa-reader at 550 nm. Iron standards were used for calibration.

### 3.3. Cell-Toxicity

Cell-toxicity was assayed by the release of lactate dehydrogenase (LDH). After the incubation with the iron oxide nanoparticles, the medium was removed, and the particles and cell debris were separated by centrifugation. Aliquots were mixed with equal amounts of LDH assay substrate, cofactor and dye solutions (each time 0.02 mL; *in vitro* toxicology assay kit lactate dehydrogenase based, Sigma, Saint Louis, U.S.). The assay mixture was added to 0.12 mL medium and incubated for 25 min in darkness. The reaction was stopped with the aid of 0.02 mL 1 M hydrochloric acid (HCl). The resulting colored compound was measured photometrically at a wavelength of 490 nm.

### 3.4. Visualization by Prussian Blue Staining and Electron Microscopy

Cells, at a confluence of 80%, were incubated with iron oxide nanoparticles in medium for 24 h. Thereafter, the cells were washed briefly twice in PBS and fixed with ice-cold acetone for 10 min. After washing in PBS, samples were stained with a filtrated 2% potassium ferrocyanide II/1 M hydrochloric acid mixture (ratio 1:1) for 10 min at 37 °C causing a deep blue complex. Then, cells were washed with PBS and subsequently counterstained with a 0.1% (w/v) solution of Nuclear Fast Red (Chroma, Stuttgart, Germany) in distilled water with 5% aluminium sulfate, for 1 min. After washing with distilled water, cells were embedded in Aquatex (Merck, Darmstadt, Germany).

For electron microscopy, cells were grown to 80% confluence on glass slides, incubated with nanoparticles in medium for 24 h, washed briefly with PBS and fixed in 3% glutaraldehyde in PBS, pH 7.2–7.4 overnight. The cells were washed with PBS six times over a period of 2 h, then stained with 2% osmium tetroxide (dissolved in distilled water) for 30 min. Then the samples were dehydrated with increasing concentrations of ethanol and embedded in araldite [10]. The hardening lasted 4 days at 37 °C and an additional 1 day at 65 °C. We obtained images on a Zeiss EM 900 electron microscope.

### 3.5. Magnetic Resonance Imaging (MRI)

Different cell numbers were cultured as monolayers on polyethylene terephthalate (PET) membrane filters (VWR International GmbH, Darmstadt, Germany) in medium, incubated with different iron oxide nanoparticles for 24 h, washed twice with PBS and fixed with 4% paraformaldehyde (dissolved in PBS) for 20 min. The iron particles caused a signal drop in T2*-weighted MRI. All MR images were obtained with a 3 T clinical scanner (Philips, Achieva). A T2*-weighted pulse sequence with TR/TE = 1000/35, a flip angle of 50°, a spatial resolution of 0.15 mm in plane, a slice thickness of 1.5 mm and four repetitions was used.

## 4. Conclusions

Contrast agents for Magnetic Resonance Imaging (MRI) improve the contrast of MRI images. One class of contrast agents are supraparamagnetic iron oxide particles (SPIOs) that produce a strongly varying local magnetic field and consequently enhance T2* relaxation. In this study, we investigated the biological properties of different nano-and micron-sized SPIOs for cellular and molecular imaging in two cell lines. The contrast agents Resovist, B102, non-coated Fe_3_O_4_ and microspheres showed a high non-specific phagocytic uptake by tumors and by endothelial cells. They are therefore well suited either for *in vitro* labeling of cells and subsequent cell tracking experiments, or for labeling of phagocytic cells *in vivo.* Resulting from our experiments, an optimized protocol for unspecific labeling could be developed: Iron uptake was most efficient with an iron concentration of 0.3 mg/mL in the cell culture medium and an incubation period of 24 h. The highest iron uptake was found for the microspheres; it was comparable to the uptake of Resovist included into liposomes or of Resovist in the presence of protamine sulfate (as reported earlier by us [11]).

The influence of size, charge and concentration of other types of SPIOs on their uptake has also been investigated earlier with the human Jurkat T cell line [12]. In agreement with our results, the dose-dependency was non-linear and independent of the particle size. Apparently, charge and coating of the particles have much larger influence on their non-specific uptake than their size.

Long residence times of the particles inside the cells allow extended imaging procedures and are therefore an important criterion [13]. Our results show that most particles remain in the cells for at least two weeks after incubation, but differences in the release kinetics are obvious between the different particles. The slowest particle release was observed for Resovist. This high retention of Resovist in comparison to other particles (not tested here) was also reported in mesenchymal stem cells [14,15]. Depending on the application, cells labeled with micron-sized particles might show a different contrast over time in dividing cells: The fewer number of particles per cell might lead to a more uneven distribution of contrast among daughter cells. All investigated particles showed a low cellular toxicity with regard to LDH release. The toxicity of Resovist, a clinically approved contrast agent, does not appear to deviate substantially from the toxicity of the other particles analyzed.

In contrast to these particles, N20, N70 and N100 Nanomag particles demonstrated a completely different iron uptake nature. These particles were hardly assimilated in any experiment, independent of the cell line used. These Nanomag particles vary in size from 20 nm to 100 nm, but this attribute seems to be insignificant. It is more probable that the coating of these particles is determining their iron uptake [16]. Therefore, these particles in combination with an appropriate coating are useful for specific labeling of molecular targets provided that they are not taken up by liver, spleen or kidneys *in vivo*.

Cells grown on polyethylene terephthalate (PET) membrane filters allow an investigation of cell monolayers by MRI, since neither the membranes nor the cell layers (even after being incubated with the nanoparticles and washed) *per se* yield a contrast. Our MRI studies with these filters confirmed the quantitative chemical determination of cell-incorporated iron. By applying Resovist to a different number of cells, we determined the sensitivity of this membrane-monolayer method for MRI visualization. Already, a cell number of 5000 (or 63 ng Fe) is enough for a clear difference to the control membrane filters.

In summary, the different iron oxide nanoparticles investigated demonstrated differential iron uptake, which can be used for diverse applications: Nanoparticles with a high non-specific uptake (microspheres, Resovist, B105, non-coated Fe_3_O_4_) are ideal for *in vitro* labeling of cells and subsequent cell-tracking or visualization of phagocytic cells *in vivo*, Nanomag particles with meager non-specific uptake are a starting point for specific labeling of molecular targets (molecular imaging).

## Figures and Tables

**Table 1:** Properties and suppliers of different iron oxide particles.

Particle	Core	Coating	Hydrodynamic Diameter	Supplier
Resovist	Maghemite/magnetite	Carboxydextran	60 nm	Schering (Berlin, Germany)
B102	Maghemite/magnetite	Carboxymethyldextran	60 nm	Dr. Norbert Buske (Berlin, Germany)
Non-coated Fe_3_O_4_	Magnetite	None	10 nm	Dr. Norbert Buske (Berlin, Germany)
Microspheres (M310)	30–60% Ferrite	Polystyrene	310 nm	Merck Estapor (Fontenay Sous Bois, France)
Microspheres (M850)	55–65% Ferrite	Polystyrene	850 nm	Merck Estapor (Fontenay Sous Bois, France)
Nanomag20 (N20)	Magnetite	Dextran	20 nm	Micromod (Rostock, Germany)
Nanomag70 (N70)	Maghemite	Dextran	70 nm	Micromod (Rostock, Germany)
Nanomag100 (N100)	Maghemite	Dextran	100 nm	Micromod (Rostock, Germany)
